# A single-donor proof-of-concept single-cell analysis maps heterogeneous differentiation trajectories toward cartilage-like states in human urine-derived stem cells

**DOI:** 10.1186/s13287-026-05223-x

**Published:** 2026-08-12

**Authors:** Alexander Schulz, Emily M. Brockmann, Miriam Zentgraf, Andreas S. Baur, Steffen Uebe, Arif B. Ekici, Mark Dedden, Sebastian Zundler, Christian T. Thiel

**Affiliations:** 1 Institute of Human Genetics, University Hospital Erlangen, Friedrich-Alexander-Universität Erlangen-Nürnberg FAU, Kussmaulallee 4, 91054 Erlangen, Germany; 2 Department of Dermatology, University Hospital Erlangen, FAU Erlangen-Nürnberg, Erlangen, Germany; 3 Department of Medicine 1, University Hospital Erlangen and Friedrich-Alexander-University Erlangen-Nürnberg, Erlangen, Germany; 4 Deutsches Zentrum Immuntherapie (DZI), University Hospital Erlangen, Erlangen, Germany

## Abstract

**Background:**

Urine-derived stem cells (USCs) represent an accessible and non-invasive cell source with reported chondrogenic differentiation potential. However, the cellular heterogeneity and transcriptional dynamics underlying USC differentiation remain incompletely understood, limiting their translational interpretation.

**Methods:**

We combined functional differentiation assays with single-cell RNA sequencing to characterize USC differentiation at both phenotypic and transcriptional levels. Chondrogenic and osteogenic differentiation were assessed using histological staining, quantitative PCR, and three-dimensional spheroid cultures. Single-cell transcriptomic analysis was performed on integrated datasets of undifferentiated and differentiated USCs, followed by pseudotime trajectory inference and mapping to a human cartilage reference atlas.

**Results:**

Chondrogenic induction resulted in reproducible acquisition of cartilage-associated features, including glycosaminoglycan-rich extracellular matrix deposition, increased expression of *SOX9*, and formation of aggrecan-positive spheroids. In this donor, single-cell analysis mapped an inferred differentiation trajectory from proliferative states towards differentiated populations, although the fine-grained pseudotemporal ordering was sensitive to analytical choices and is therefore interpreted qualitatively. Along this inferred trajectory, we identified a candidate transient transcriptional state associated with elevated *CDH1* expression and epithelial-like aggregation features. Probabilistic mapping to a human cartilage reference atlas indicated that overall mapping confidence was low (median prediction score 0.34) and that only a minority of cells showed confident transcriptional similarity (prediction score ≥ 0.5) to mature/articular cartilage-associated reference states (7.8% of all cells and 17.6% of chondrogenically induced cells). This confident similarity was concentrated in a few clusters at the differentiated end of the trajectory rather than representing the bulk of the culture, and label-transfer confidence was not equated with chondrocyte identity. Despite this enrichment, differentiated populations exhibited transcriptional heterogeneity, including subsets of cells associated with hypertrophic, fibrocartilage-like, and contractile gene programmes, indicating the presence of multiple differentiation trajectories.

**Conclusions:**

This single-donor proof-of-concept study suggests that USC differentiation may involve a candidate transient, aggregation-associated transcriptional state accompanied by *CDH1* expression and gives rise to heterogeneous lineage-associated outcomes, with only a minority of cells acquiring confident transcriptional similarity to mature cartilage. Because these observations derive from one donor, they should be interpreted as hypothesis-generating and require validation across independent donors before donor-independent or translational conclusions for cartilage regeneration can be drawn. These findings nonetheless provide a single-cell resolution framework for future multi-donor validation of USC differentiation and its inherent transcriptional heterogeneity.

**Graphical Abstract:**

**Figure:**
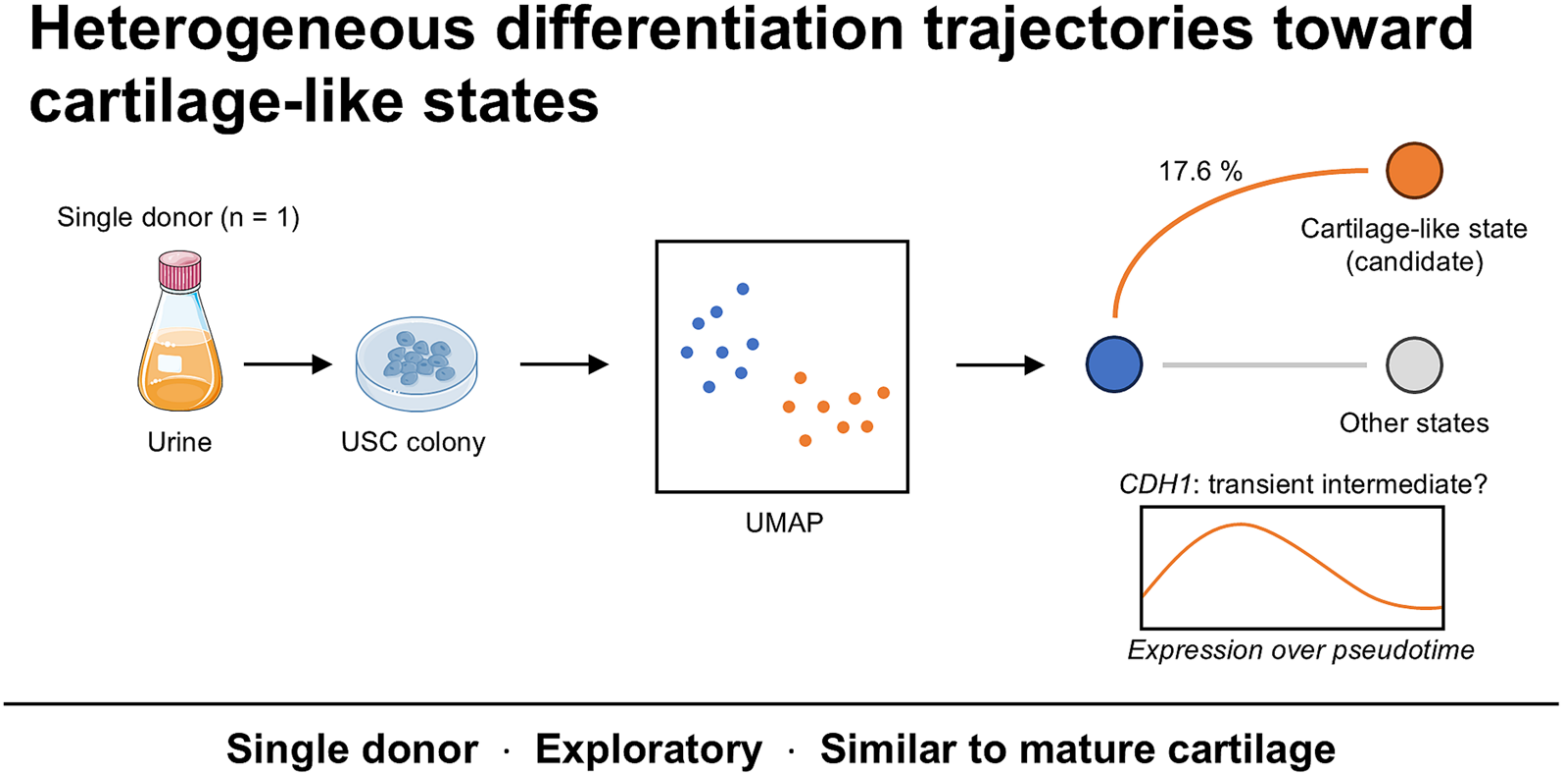

**Supplementary Information:**

The online version contains supplementary material available at 10.1186/s13287-026-05223-x.

## Background

Understanding human cartilage development and its dysregulation in skeletal disorders remains a major challenge, largely due to the limited accessibility of primary human cartilage tissue [1–3]. Functional studies of chondrogenesis often rely on mesenchymal stem cells (MSCs) or induced pluripotent stem cells (iPSCs), which require invasive sampling or complex reprogramming procedures and may not fully recapitulate physiological differentiation processes [4–7].

Urine-derived stem cells (USCs) have emerged as an attractive cell source due to their non-invasive accessibility and robust expansion capacity [8–10]. Previous studies have demonstrated multilineage differentiation potential in USCs, including chondrogenic differentiation [11, 12]. However, the cellular composition of cultured USCs and the transcriptional programs underlying their differentiation remain incompletely understood [11, 13, 14]. In particular, it remains unclear whether USC-derived cells follow structured differentiation trajectories and to what extent they resemble defined cartilage-associated transcriptional states. Moreover, inter-individual variability in USC composition and differentiation potential has been reported, highlighting the need for careful interpretation of findings derived from individual samples [13–15].

Recent advances in single-cell RNA sequencing (scRNA-seq) enable high-resolution dissection of cellular heterogeneity and lineage progression [16, 17]. These approaches provide the opportunity to map differentiation processes at the level of individual cells and to identify transitional states and regulatory programs [18]. However, to our knowledge, such analyses have not been systematically applied to USC chondrogenesis. These approaches are particularly relevant for systems such as USC differentiation, where cellular heterogeneity and intermediate states are not resolved by bulk analyses.

In addition, the translational potential of USCs depends on clinically compatible culture conditions. Conventional protocols rely on animal-derived components such as fetal bovine serum (FBS), which limit reproducibility and clinical applicability [19–21]. Xeno-free culture systems based on autologous human serum (HS) represent a potential alternative, but their impact on USC differentiation trajectories and transcriptional states remains insufficiently characterized [22, 23]. In addition, variability in serum composition may affect reproducibility and scalability, which are important considerations for clinical translation [24].

Here, we present a single-donor proof-of-concept single-cell analysis of USC chondrogenesis, combined with functional differentiation assays. We define the cellular heterogeneity of cultured USCs in this donor, infer candidate differentiation trajectories, and assess their transcriptional relationship to cartilage-associated states. Furthermore, we evaluate the effects of xeno-free culture conditions on differentiation outcomes. The study provides a framework for future multi-donor validation of USC chondrogenesis at single-cell resolution and highlights both cartilage-associated and alternative transcriptional programmes arising during differentiation. These findings underscore the importance of resolving intermediate and heterogeneous states when interpreting stem cell differentiation systems [25–27].

## Methods

### Urine-derived stem cell isolation and culture

Urine-derived stem cells (USCs) were isolated and expanded using a modified urine-cell culture protocol [28, 29]. Briefly, urine samples were centrifuged at 300 × g, the pellet was washed in phosphate-buffered saline (PBS) containing 1% penicillin/streptomycin (Gibco), and recovered cells were seeded for culture after repeated centrifugation. Standard expansion was performed in a 1:1 mixture of DMEM high glucose (Gibco), supplemented with 1% non-essential amino acids and 1% penicillin/streptomycin (Gibco), and keratinocyte serum-free medium (KSFM, Gibco), supplemented with 15% fetal bovine serum (FBS; Sigma-Aldrich or Roth) and 5 ng/mL FGF2 (PeproTech or thermostable humankine FGF2, Sigma). Medium was changed three times per week, and no cultures beyond passage 5 were used for experiments.

For exploratory xeno-free culture experiments, pelleted urinary cells were resuspended in Alpha MEM Eagle medium (PAN Biotech) supplemented with 5 ng/mL FGF2 (Sino Biological) and either 10% FBS or 10% autologous human serum (HS), and 1% penicillin/streptomycin.

### Chondrogenic and osteogenic differentiation

Chondrogenic and osteogenic differentiation were performed as described previously [29]. For monolayer chondrogenic differentiation, USCs were cultured for 2 weeks using the StemPro Chondrogenesis Differentiation Kit (Gibco), and 1% penicillin/streptomycin (Gibco). Osteogenic differentiation was induced for 2 weeks using an osteogenic medium based on a published formulation [30]. Media were changed three times per week. To visualize lineage-associated matrix deposition, cells were fixed in 4% paraformaldehyde and stained with Alcian blue for glycosaminoglycans or Alizarin red S for mineral deposition. As undifferentiated controls, matched USC cultures from the same donor were maintained for 2 days in Alpha MEM Eagle medium supplemented with 10% FBS and 5 ng/mL FGF2, and 1% penicillin/streptomycin.

For comparative pellet differentiation under HS and FBS conditions, 250,000 USCs were centrifuged at 300 × g for 10 min in 15 mL tubes (Corning) and cultured in an in-house chondrogenic medium consisting of DMEM high glucose with pyruvate (Gibco), recombinant human insulin (6.25 μg/mL; Merck), holo-transferrin (6.25 μg/mL; Sigma-Aldrich), sodium selenite (6.7 ng/mL; Sigma-Aldrich), 10% serum (autologous HS or FBS), recombinant human TGF-β1 (10 ng/mL; PeproTech), recombinant human IGF1 (100 ng/mL), dexamethasone (100 nM; Sigma-Aldrich), and ascorbate-6-phosphate (50 μg/mL; Sigma-Aldrich), and 1% penicillin/streptomycin. Alcian blue quantification was performed after lysis for 2 h at room temperature in 6 M guanidine hydrochloride (Sigma-Aldrich), followed by absorbance measurement at 650 nm. DNA-based normalization was performed using parallel control samples processed with the DNeasy Blood & Tissue Kit (Qiagen).

### Three-dimensional spheroid culture and aggrecan immunofluorescence

Chondrogenic spheroid differentiation and staining were performed as described previously [29]. For three-dimensional (3D) chondrogenic culture, 20,000 USCs were seeded into each well of a 96-well Nunclon Sphera ultra-low attachment plate (Thermo Fisher Scientific) and centrifuged for 10 min at 300 × g to initiate scaffold-free aggregation. Spheroids were cultured for 3 weeks using the StemPro Chondrogenesis Differentiation Kit, and 1% penicillin/streptomycin. Undifferentiated controls consisted of matched USC cultures maintained for 2 days in Alpha MEM Eagle medium containing 10% FBS and 5 ng/mL FGF2.

For aggrecan immunofluorescence, spheroids were fixed for 1 h in 4% paraformaldehyde at room temperature, permeabilized with Triton X-100 for 30 min, and blocked in 1% bovine serum albumin (BSA) in PBS for 45 min. Samples were incubated with anti-aggrecan antibody (Abcam, catalog no. ab3778, 1:500), followed by Alexa Fluor 488-conjugated anti-mouse secondary antibody (Invitrogen, catalog no. A28175, 1:500) together with DAPI (1:1000; Thermo Fisher Scientific). After washing, spheroids were mounted in Aqua-Polymount (Polysciences) on concave slides (Paul Marienfeld). Imaging was performed using an Axio Imager 2 with Apotome (Zeiss) using 25 optical sections. Exposure times were kept constant between differentiated and undifferentiated spheroids (DAPI: 10 ms; aggrecan: 35 ms), and identical image-enhancement settings were applied across conditions using ImageJ/FIJI [31].

### E-cadherin immunofluorescence

For E-cadherin immunofluorescence, independently cultured USC samples from three donors after 5 days of chondrogenic induction were analyzed. Undifferentiated cells were maintained on 0.2% gelatin-coated culture vessels in Alpha MEM Eagle medium supplemented with 5 ng/mL FGF2 and 20% fetal bovine serum (FBS) and 1% penicillin/streptomycin, whereas differentiated cells were cultured on 0.2% gelatin in StemPro Chondrogenesis Differentiation Kit and 1% penicillin/streptomycin according to the manufacturer’s instructions. Cells were washed once in PBS, fixed in 4% paraformaldehyde for 30 min at room temperature, air-dried for 30 min, washed twice in PBS, and incubated in 0.01% Tween-20 in PBS for 10 min. After two additional PBS washes, samples were blocked for 60 min at room temperature in blocking solution. Primary antibody incubation was performed overnight at 4 °C in blocking solution using mouse anti-human E-cadherin monoclonal antibody HECD-1 (Thermo Fisher Scientific, catalog no. 13-1700, 1:500). Samples were then washed three times in PBS and incubated with Alexa Fluor 488-conjugated anti-mouse secondary antibody (Invitrogen, catalog no. A28175, 1:500) together with DAPI (1:1000; Thermo Fisher Scientific, catalog no. 62248) for 2 h at room temperature in the dark, followed by three PBS washes, a final rinse in distilled water, and mounting in Aqua-Polymount. Imaging was performed using an Axio Imager 2 with Apotome (Zeiss) using 3 optical sections. Exposure times were kept constant between differentiated and undifferentiated cells (DAPI: 60 ms; E-cadherin: 600 ms). Image enhancement was performed in ImageJ/FIJI [31]. Z-stacks were combined by maximum intensity projection. To account for differences in cell density and acquisition conditions, a linear normalization factor was determined per image based on the mean DAPI intensity within Otsu-thresholded nuclear regions. This factor was applied equally to both DAPI and CDH1 channels. Identical linear display settings were then applied to all images within the figure panel. Adjustments were used for visualization only.

### Reverse transcription quantitative PCR

RT-qPCR was performed as described previously [29]. Total RNA was isolated from USC samples using either the RNeasy Mini Kit (Qiagen) with on-column DNase digestion or, where applicable, the NucleoSpin RNA Plus Kit (Macherey-Nagel) with genomic DNA removal. Samples intended for direct comparison were processed using the same extraction workflow. cDNA was synthesized using LunaScript RT SuperMix (New England Biolabs). RT-qPCR was performed using TaqMan Master Mix and predesigned TaqMan assays (Applied Biosystems) on a QuantStudio 12 K Flex platform (Applied Biosystems). *RPLP0* served as the housekeeping gene. Relative expression was calculated using the ΔΔCt method from four technical replicates. Statistical analyses were based on unpaired Welch’s t-tests applied to ΔΔCt values. Three biological replicates per condition were used.

### Bulk RNA sequencing and differential expression analysis

Bulk RNA sequencing was performed as described previously [29]. Bulk RNA was extracted using the RNeasy Mini Kit (Qiagen) with DNase digestion. Stranded mRNA libraries were generated using the Illumina Stranded mRNA kit and sequenced as paired-end reads with a mean fragment length of 159 bp on an Illumina NovaSeq 6000 platform. Raw sequencing output was demultiplexed using Illumina Dragen software (v3.8.4). Unwanted RNA species were removed using bwa mem (v0.7.17-r1188) in combination with samtools (v1.17) [32], and filtered reads were converted back to FASTQ using SamToFastq from GATK (v4.2.1.0) [33]. Reads from each lane were aligned individually to the hg38 reference genome with Ensembl [34] release 110 gene annotations using STAR (v2.7.10a) [35]. Lane-level alignments were merged with Picard MergeSamFiles (v2.25.4), and gene-level counts were generated using featureCounts (v2.0.1) [36]. Alignment and quantification metrics were used for quality assessment.

Differential expression analysis was performed in R (v4.5) using DESeq2 (v1.48.1) [37]. For each comparison, gene-level counts were used to construct a DESeqDataSet object with condition labels defining the experimental groups. Differential expression was calculated with DESeq(), and contrasts were specified so that positive log2 fold changes represented upregulation in the differentiated condition relative to undifferentiated controls. Genes with Benjamini-Hochberg adjusted *P* values < 0.05 were considered significant. Gene identifiers were mapped to HGNC symbols using org.Hs.eg.db (v3.21.0) [38]. For exploratory sample-level visualization, regularized-log-transformed count matrices were variance-filtered and analyzed by principal component analysis using prcomp(). Volcano plots were generated with ggplot2 (v4.0.1) [39] and ggrepel (v0.9.6) [40].

### Single-cell RNA sequencing

Single-cell RNA-sequencing samples were processed as described previously [29]. Undifferentiated and chondrogenically differentiated USC samples were processed for single-cell RNA sequencing using the Parse Biosciences Evercode WT v3 workflow, including fixation, barcoding, cDNA amplification, and library generation. Libraries were sequenced on an Illumina NovaSeq X Plus. FASTQ files were processed using the Parse Biosciences Trailmaker pipeline (v1.5.0), and the resulting count matrices were imported into Seurat (v5) [41] for downstream analysis.

### Preprocessing of uUSC and chUSC datasets

Initial preprocessing of undifferentiated and differentiated datasets was performed separately. For each object, mitochondrial content was calculated using PercentageFeatureSet. Cells were filtered using sample-specific thresholds defined from the 1st and 99th percentiles of nFeature_RNA and nCount_RNA, together with the 95th percentile of mitochondrial reads. The filtered uUSC and chUSC datasets were then log-normalized, variable genes were identified using the vst method (nfeatures = 3000), scaled while regressing only percent.mt, and analyzed by principal component analysis (PCA), nearest-neighbor graph construction, Louvain clustering, and uniform manifold approximation and projection (UMAP). PCA dimensions 1–20 and clustering resolution 0.5 were used for both datasets. Cluster markers were identified using FindAllMarkers with only.pos = TRUE, min.pct = 0.25, and logfc.threshold = 0.25. Transcript count (nCount_RNA) was not regressed in the revised analysis pipeline.

### Panel-based annotation of uUSC states

To annotate uUSC transcriptional states, curated marker panels representing renal epithelial/parietal epithelial cell (PEC)-associated, proximal tubule-like (PT), distal thin limb-like (DTL), thick ascending limb-like (TAL), urothelial, stromal fibroblast-like, smooth muscle/contractile, and cycling programmes were assembled from established renal and urinary markers [42–46]. Module scores were calculated using AddModuleScore, retaining only panels with at least three genes present in the dataset. Median module scores were summarized per cluster and visualized by heatmaps and dot plots.

### Cell cycle scoring and CytoTRACE2 analysis

Cell cycle phase scores were computed using CellCycleScoring with Seurat’s built-in S-phase and G2/M gene sets. A combined cycling score was calculated as the row mean of the S and G2/M scores.

CytoTRACE2 (v1.1.0) [47] was applied to the uUSC dataset to estimate relative developmental potential. For memory-efficient execution, the top 3,000 variable genes from the RNA counts layer were retained, and cells were downsampled in a stratified manner to a maximum of 300 cells per cluster. CytoTRACE2 was then run on the dense count matrix of the sampled subset. To extend scores to all cells, CytoTRACE2 values from sampled cells were projected to the full dataset using k-nearest-neighbor averaging (k = 15) in PCA space (PCs 1–20) with the FNN package. The projected score was used for downstream visualization and interpretation.

### Construction of the cartilage reference atlas

A cartilage reference atlas was assembled from publicly available single-cell data from normal human cartilage samples in the Huang et al. dataset [48]. Six normal cartilage samples (GSM6797148_CartNorm1 to GSM6797153_CartNorm6) were imported with ReadMtx, and Seurat objects were created using min.cells = 3 and min.features = 200. Cells were filtered using nFeature_RNA ≥ 300, nCount_RNA ≥ 500, and percent.mt < 15. To maintain balanced sample representation, at most 4,000 cells per sample were retained by random downsampling. Each sample was SCTransformed while regressing percent.mt and using 3,000 variable features. Integration was performed with SelectIntegrationFeatures, PrepSCTIntegration, FindIntegrationAnchors, and IntegrateData using SCT normalization and 30 dimensions. The integrated reference atlas was then processed by PCA, neighbor graph construction, clustering at resolution 0.5, and UMAP. Reference cluster markers were calculated using FindAllMarkers on the SCT assay.

To derive biologically interpretable cartilage states, the reference atlas was additionally scored using curated chondrocyte gene panels representing homeostatic/articular, mature chondrocyte, hypertrophic, fibrochondrocyte, and proliferative programmes. State assignments were based on the dominant module score across these panels [46, 49–52].

### Integration of uUSC and chUSC datasets

For the revised joint analysis, uUSC and chUSC Seurat objects were integrated rather than simply merged. Each dataset was normalized and processed in parallel, variable genes were identified (nfeatures = 3000), and shared integration features were selected using SelectIntegrationFeatures. After feature-restricted scaling and PCA, integration anchors were computed using reciprocal PCA (reduction = "rpca", dims = 1:30), and an integrated assay was generated with IntegrateData. The integrated object was subsequently scaled and analyzed using PCA, nearest neighbors, Louvain clustering (resolution 0.6), and UMAP based on dimensions 1–30.

### Reference mapping of integrated USC states

For cartilage-state transfer, both the integrated USC object and the cartilage reference were analyzed on the RNA assay. Both objects were log-normalized and processed with FindVariableFeatures (nfeatures = 3000). Shared transfer features were defined as the intersection of variable genes present in both objects. The reference object was scaled on these shared genes and an RNA-based PCA reduction was calculated specifically for transfer. Label transfer anchors were generated with FindTransferAnchors using LogNormalize-based transfer and the reference PCA reduction (dims = 1:30), and reference cluster labels were projected to the USC dataset with TransferData. Per-cell transferred identities and maximum prediction scores were stored as PredictedRefCluster and PredictionScore, respectively. Mapping was evaluated probabilistically rather than as a categorical identity. The full distribution of per-cell prediction scores was summarized overall and by stage and cluster, and the fraction of cells exceeding prediction-score thresholds of 0.4, 0.5 and 0.6 was reported; 0.5 was used as the primary cutoff (corresponding to a majority anchor vote for one reference class) and 0.4/0.6 as sensitivity bounds. Reference clusters were assigned to cartilage states by their dominant curated subtype module score, and a cell was considered to show confident similarity to mature/articular cartilage when its best-matching reference cluster belonged to the mature/articular group and its prediction score was ≥ 0.5. Named cartilage-state percentages were not reported, because best-label assignment of each cell to a single named cartilage state would overstate categorical identity at the observed low mapping confidence; mapping was instead summarised probabilistically using per-cell prediction scores.

### Lineage-associated state scoring in the integrated USC object

To characterize differentiated cell-state heterogeneity, the integrated USC object was further scored with curated lineage-associated marker panels representing hypertrophic, fibrocartilage-like, and smooth muscle/contractile programmes. These module scores were used for feature visualization and for comparison of alternative differentiation-associated transcriptional programmes across clusters and pseudotime-relevant states. The cartilage-state signatures used in the late-cluster dot plots included homeostatic/articular, mature chondrocyte, hypertrophic, fibrochondrocyte, smooth muscle/contractile, and mesenchymal gene sets [46, 49–51].

### Pseudotime inference

Pseudotime analysis was performed on the integrated, state-scored USC object using Slingshot (v2.16.0) [53]. The integrated Seurat object was slimmed to the RNA assay and UMAP embedding, converted to a SingleCellExperiment, and supplied to Slingshot using UMAP coordinates as the reduced dimension and Seurat cluster labels as the cluster annotation. The root cluster was selected from uUSC-dominant proliferative clusters based on high *TOP2A* expression together with elevated CytoTRACE2 values. Slingshot was run with omega = TRUE, and pseudotime values were extracted from the lineage matrix. When multiple lineages were returned, a per-cell mean pseudotime was calculated across lineages. Pseudotime summaries were then calculated by stage and cluster.

To assess whether the inferred trajectory reflected differentiation rather than a mixture of cell types, pseudotime was re-evaluated after excluding non-epithelial, off-trajectory clusters (smooth muscle-, myofibroblast-, and neuronal-like clusters; integrated clusters 5, 10 and 12). The remaining epithelial-derived subset (23,242 cells) was re-processed from counts using the same parameters as the integrated workflow (re-normalization, variable-feature selection with nfeatures = 3000, reciprocal-PCA integration with dims = 1:30, re-clustering at resolution 0.6 and UMAP on dimensions 1–30), and Slingshot was re-run on the new embedding with the root again selected from the cycling, uUSC-dominant cluster. Robustness of the resulting pseudotemporal ordering was quantified by Spearman and Pearson correlation of per-cell and cluster-median pseudotime against the original trajectory and against an independent subset analysis performed on the existing embedding. The cluster definitions, marker evidence and robustness metrics underlying this reanalysis are provided in Additional file 4 and Additional file 1 (Supplementary Fig. S4).

### Marker analysis along pseudotime

To identify genes associated with early, intermediate, and late integrated states, marker analysis was performed on the pseudotime-annotated integrated object using FindAllMarkers on the RNA assay with only.pos = TRUE, min.pct = 0.15, logfc.threshold = 0.25, and return.thresh = 0.05. Cluster-level pseudotime summaries were calculated from median pseudotime values per cluster, and clusters were assigned to coarse early, mid, and late bins using tertile-based cutoffs of median pseudotime. Top markers per cluster were linked to their pseudotime summaries, and candidate intermediate markers were ranked based on recurrence across mid-pseudotime clusters. These analyses informed the marker sets shown in the focused and supplementary pseudotime-ordered dot plots. A complete overview of the top cluster markers in the integrated dataset, linked to pseudotime bins, is provided in Supplementary Table S1 (Additional file 2). Complete per-cluster differentially expressed gene lists, explicit cluster annotations, and the full gene composition of all marker panels shown in the figures are provided in Additional file 4. *CDH1* dynamics were examined on the re-integrated epithelial subset by quantifying mean *CDH1* expression and the percentage of *CDH1*-positive cells across pseudotime deciles, integrated clusters and stage, with *TOP2A* analysed in parallel as a proliferation-associated early marker; monotonic association with pseudotime was summarized by Spearman correlation.

### Statistical analysis and visualization

Statistical analyses for qPCR and selected wet-lab assays were performed in R and GraphPad Prism v10, using unpaired Welch’s *t*-tests unless otherwise stated. Single-cell and bulk visualizations were generated in R using Seurat (v5) [41], ggplot2 (v4.0.1) [39], patchwork (v1.3.2) [54], pheatmap (v1.0.13) [55], viridis (v0.6.5) [56], and ggrepel (v0.9.6) [40].

## Results

### Cultured uUSCs comprise a heterogeneous epithelial-derived population with distinct proliferative and lineage-associated transcriptional programmes

**Fig. 1:**
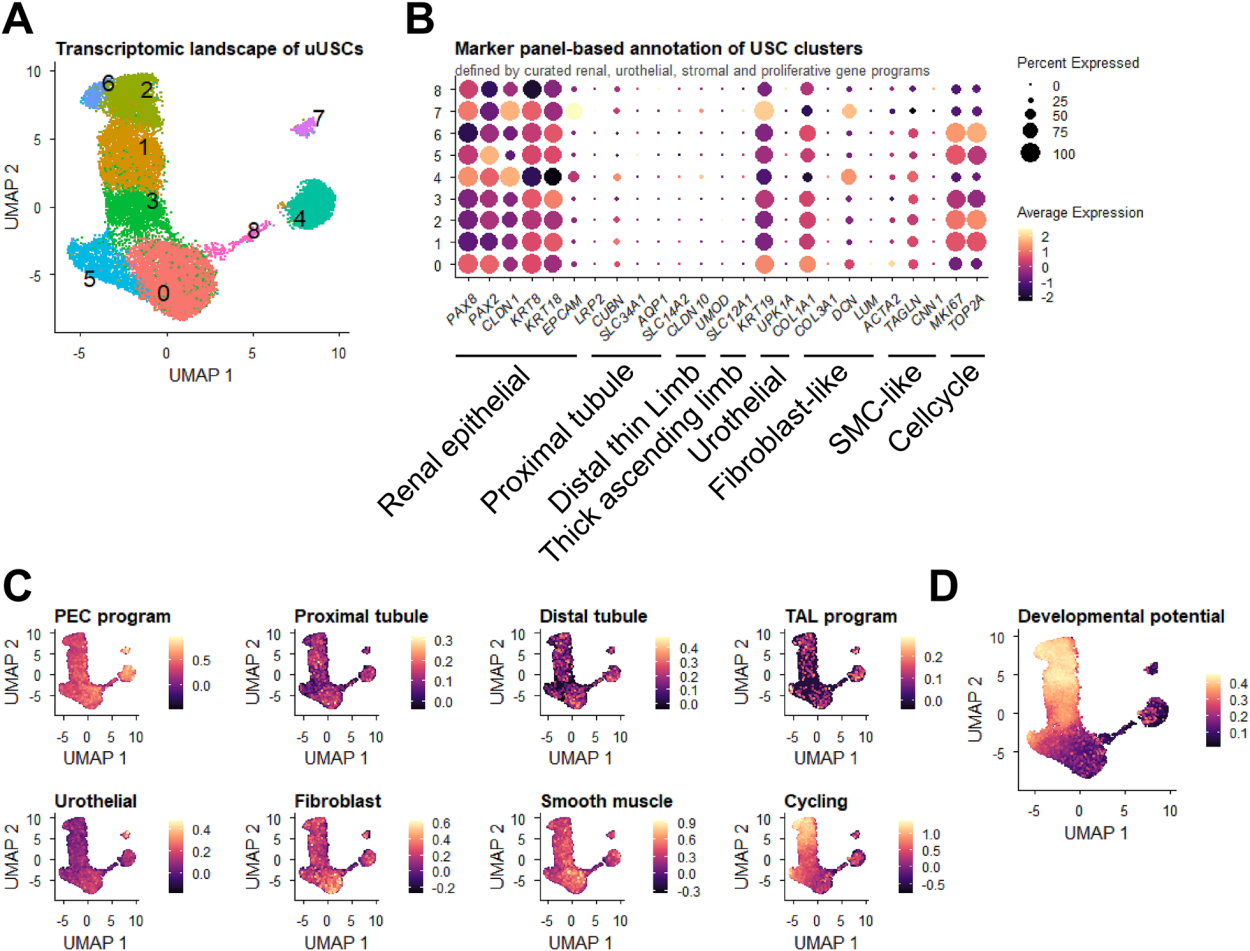
Transcriptional landscape and programme-based annotation of undifferentiated USCs. **A** UMAP of undifferentiated urine-derived stem cells (uUSCs) after quality control filtering, showing transcriptionally distinct clusters. **B** Dot plot of curated marker genes used for cluster annotation across uUSC clusters. **C** Feature plots showing module scores for renal epithelial/parietal epithelial cell (PEC)-associated, proximal tubule-like, distal tubular, thick ascending limb-like, urothelial, stromal fibroblast-like, smooth muscle/contractile, and cycling programmes. **D** UMAP showing projected CytoTRACE2 scores across the uUSC manifold. CytoTRACE2 scores were interpreted as relative developmental potential rather than direct measures of stemness

To characterize the cellular composition of cultured undifferentiated urine-derived stem cells (uUSCs), we performed single-cell RNA sequencing following quality control filtering, yielding a total of 17710 cells distributed across multiple transcriptionally distinct clusters (Fig. 1A). Quality control metrics and filtering criteria are provided in Supplementary Fig. S1A, B. Cluster sizes varied, indicating non-uniform representation of cellular states within the culture.

To annotate these clusters in a robust and unbiased manner, we applied a panel-based scoring strategy using curated gene sets representing renal epithelial/parietal epithelial cell (PEC)-associated, proximal tubule-like, distal tubular, urothelial, stromal fibroblast-like, smooth muscle/contractile, and cycling programmes [42–46] (Fig. 1B, C). This approach avoids overinterpretation based on individual marker genes and instead captures broader transcriptional programmes.

Across clusters, we observed a predominant epithelial-associated compartment, with several clusters showing higher scores for renal epithelial and PEC-related programmes. In parallel, distinct subpopulations displayed elevated scores for alternative transcriptional programmes, including proximal tubule-like and urothelial signatures, as well as stromal extracellular matrix and contractile gene programmes. Notably, a subset of clusters was characterized by strong enrichment of cycling-associated genes, including *TOP2A* and *MKI67*, indicating a proliferative compartment within the cultured population.

Comparison of programme scores across clusters demonstrated that no single transcriptional identity uniformly defined all uUSCs. Cluster annotations were supported by concordant enrichment of multiple marker genes and programme-level signatures rather than reliance on individual gene expression. Instead, individual clusters were characterized by dominant but partially overlapping programmes, reflecting a structured and heterogeneous population rather than a homogeneous cell type. In particular, stromal-associated (*COL1A1*, *DCN*, *LUM*) and contractile (*ACTA2*, *TAGLN*, *CNN1*) programmes were confined to specific clusters, supporting the presence of non-epithelial transcriptional states in vitro.

To further assess relative developmental state, we applied CytoTRACE2 and visualized the inferred developmental potential across the uUSC manifold [47] (Fig. 1D). As CytoTRACE2 primarily reflects relative developmental potential and is influenced by proliferative activity in cultured cells, we interpreted these scores in the context of the identified cycling programme. Higher CytoTRACE2 values were predominantly associated with proliferative clusters, consistent with increased transcriptional diversity in cycling cells rather than definitive stemness per se. Accordingly, CytoTRACE2 scores were not interpreted as direct measures of stemness but rather as indicators of relative transcriptional diversity within the cultured population.

Together, these results define cultured uUSCs as a heterogeneous epithelial-derived population comprising proliferative, epithelial-associated, and stromal/contractile transcriptional states. This structured heterogeneity provides the foundation for analyzing differentiation trajectories and lineage-associated programmes in subsequent experiments.

### USCs exhibit reproducible lineage-associated differentiation features across targeted functional assays

**Fig. 2:**
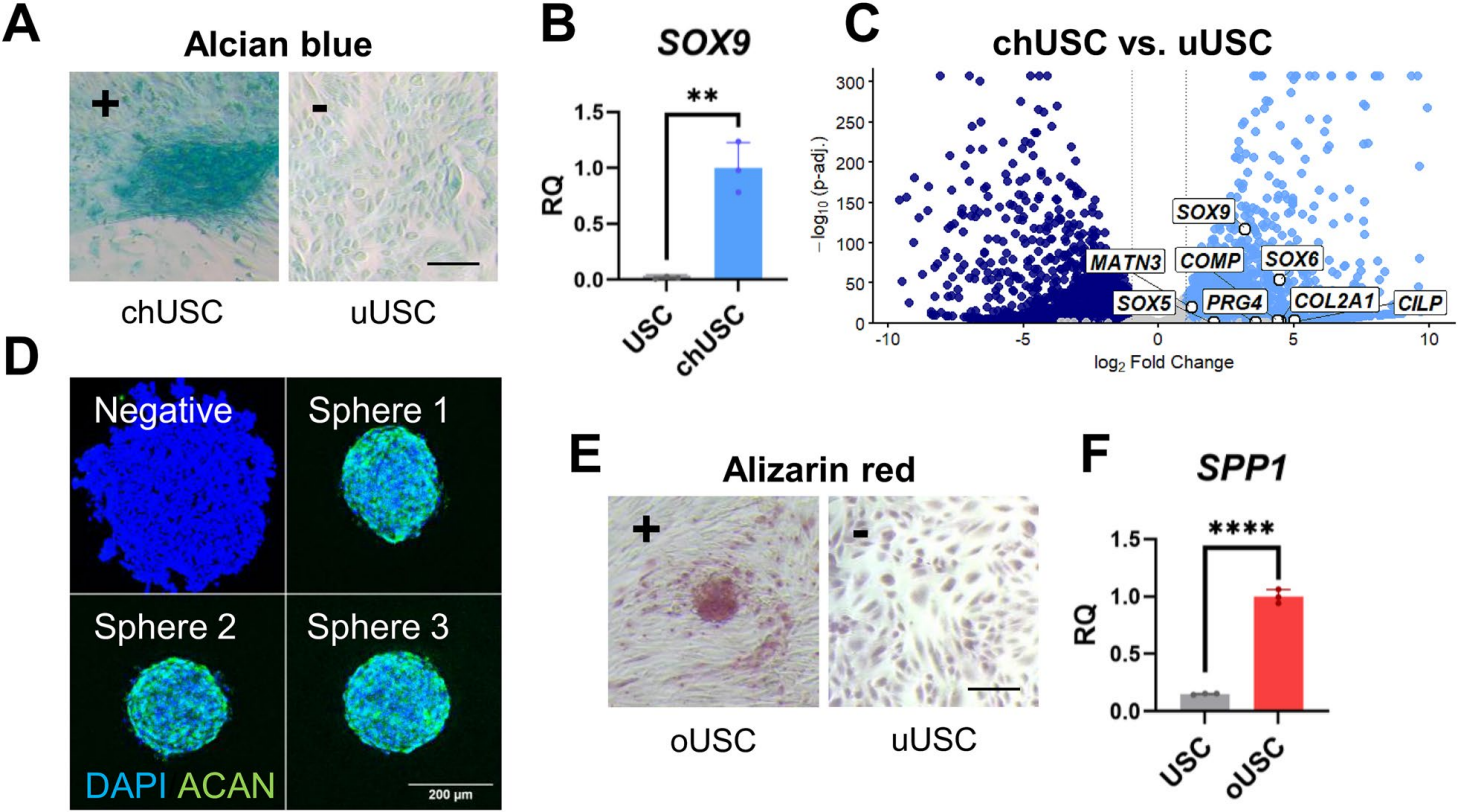
Functional lineage-associated differentiation features of USCs. **A** Alcian blue staining of chondrogenically (chUSC) differentiated and undifferentiated (uUSC) USC cultures after 2 weeks of induction. **B** RT-qPCR analysis of *SOX9* expression in undifferentiated and chondrogenically differentiated USC cultures. Relative expression values (RQ) were 0.017 ± 0.001 in undifferentiated cells and 1.00 ± 0.23 in differentiated cells (*n* = 3; *p* < 0.01, Welch’s *t*-test). Bars with undetermined Ct values or values exceeding 40 cycles were indicated as ND. **C** Volcano plot from bulk RNA-sequencing comparing undifferentiated and chondrogenically differentiated USC cultures, with selected cartilage-associated genes highlighted. Highlighted genes met the indicated thresholds for statistical significance and effect size. The selected genes shown are *SOX9, SOX5, SOX6, COL2A1, COMP, PRG4, CILP and MATN3*. The full differential expression workflow is described in Methods. **D** Immunofluorescence staining of scaffold-free spheroids showing aggrecan deposition in chondrogenically differentiated cultures and undifferentiated (negative) controls. Representative differentiated spheroids (sphere 1–3) and negative control are shown. Scale bar, 200 µm. Nuclei were counterstained with DAPI. A 3D rendering of a representative differentiated spheroid is provided in Supplementary Video S1 (Additional file 3). **E** Alizarin red staining of osteogenically differentiated (oUSC) and undifferentiated (uUSC) USC cultures after 2 weeks of induction. **F** RT-qPCR analysis of *SPP1* expression in undifferentiated and osteogenically differentiated USC cultures. Relative expression values (RQ) were 0.149 ± 0.006 in undifferentiated cells and 1.00 ± 0.06 in differentiated cells (*n* = 3; *p* < 0.0001, Welch’s *t*-test)

To functionally assess the differentiation capacity of urine-derived stem cells (USCs), we subjected cultured cells to chondrogenic and osteogenic induction and evaluated lineage-associated features using complementary assays (Fig. 2).

Chondrogenically induced cultures showed positive Alcian blue staining, indicating the presence of glycosaminoglycan-rich extracellular matrix [57] (Fig. 2A). Consistent with activation of a chondrogenic transcriptional programme, RT-qPCR analysis revealed increased expression of the key regulator *SOX9* following differentiation [58] (Fig. 2B). In addition, targeted analysis of selected canonical cartilage-associated regulators and matrix genes, including *SOX9*, *SOX5*, *SOX6*, *COL2A1*, *COMP*, and *MATN3*, demonstrated increased expression following chondrogenic induction [59–61] (Fig. 2C).

To assess matrix formation in a 3D context, we generated scaffold-free spheroids under chondrogenic conditions. Immunofluorescence analysis revealed robust deposition of aggrecan (*ACAN*), a major cartilage extracellular matrix component, in differentiated spheroids compared to undifferentiated controls [62] (Fig. 2D). This finding indicates that USCs are capable of forming cartilage-like extracellular matrix in 3D culture.

To investigate lineage specificity, we additionally evaluated osteogenic differentiation. Induced cultures showed Alizarin red–positive mineral deposition (Fig. 2E) and increased expression of the osteogenic marker *SPP1* by RT-qPCR (Fig. 2F), indicating retention of alternative mesenchymal differentiation capacity [59–64].

Together, these results show that USCs reproducibly acquire cartilage-associated molecular and extracellular matrix features under chondrogenic conditions [58, 65]. However, as extracellular matrix production is not exclusive to cartilage and may arise in other mesenchymal contexts, these findings do not exclude the presence of alternative or hypertrophic differentiation states and were therefore interpreted alongside single-cell analyses to resolve cellular heterogeneity [66, 67].

### A candidate transient *CDH1*-associated intermediate state in USC differentiation

**Fig. 3:**
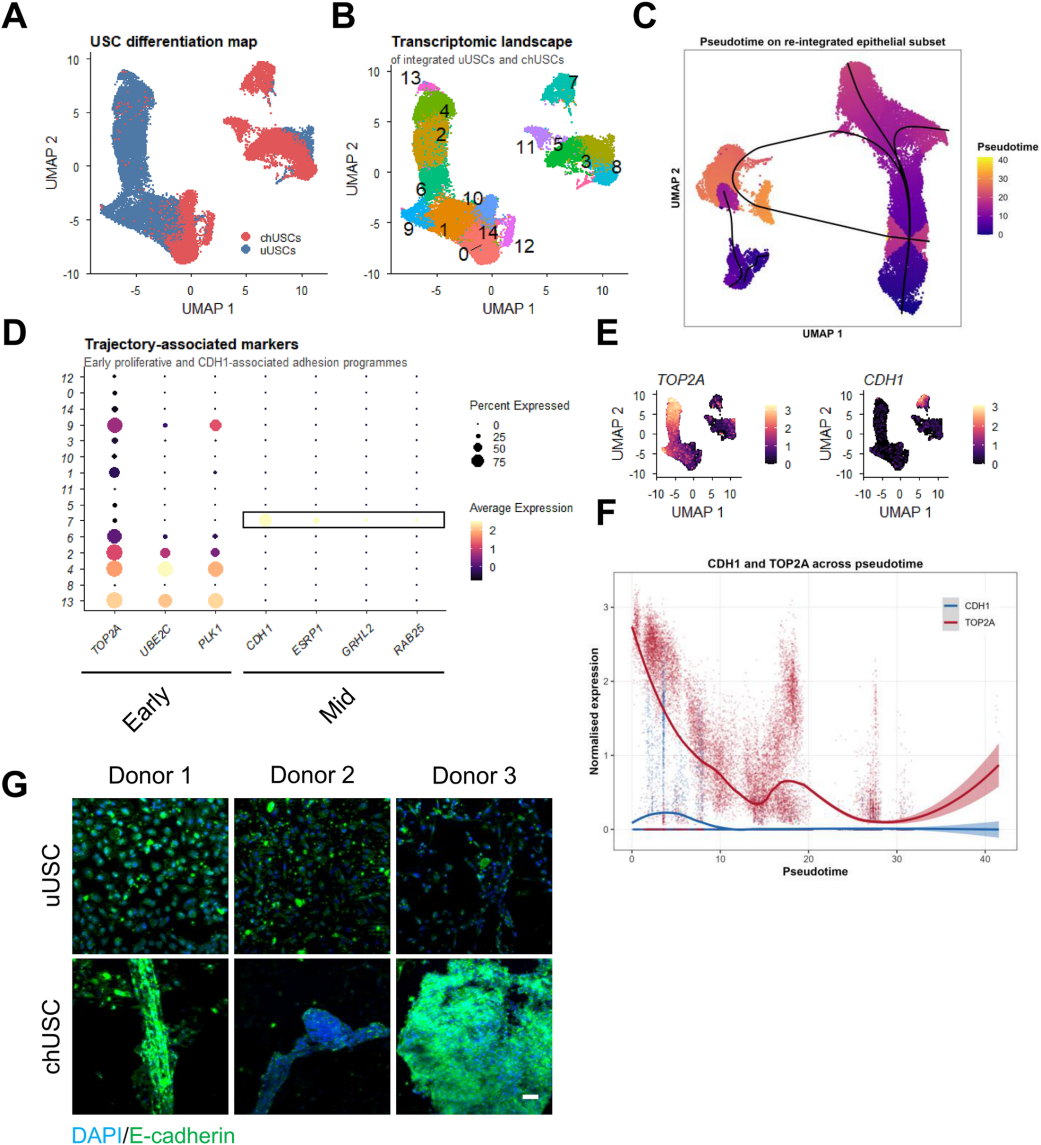
USC differentiation involves a candidate transient aggregation-associated intermediate state associated with *CDH1* expression. **A** UMAP of the integrated uUSC and chondrogenically differentiated USC dataset colored by stage. **B** UMAP of the integrated dataset colored by Seurat cluster identity. **C** Pseudotime projection of the inferred differentiation trajectory from undifferentiated towards differentiated states, computed on the re-integrated epithelial subset after exclusion of non-epithelial, off-trajectory clusters. The coarse direction is robust, whereas the fine-grained ordering is not (Additional file 1, Supplementary Fig. S4); the trajectory is interpreted qualitatively. **D** Dot plot of curated early and intermediate pseudotime-associated markers across pseudotime-ordered clusters. **E** Feature plots of *TOP2A* and *CDH1* expression in the integrated dataset. **F** Expression dynamics of *TOP2A* and *CDH1* along pseudotime. Computed on the re-integrated epithelial subset: *CDH1* rises to an early peak and decreases thereafter (candidate transient behaviour along a non-robust pseudotime axis), whereas *TOP2A* declines monotonically as an early proliferation marker (see also Additional file 1, Supplementary Fig. S2; Additional file 4). **G** Immunofluorescence staining of E-cadherin in undifferentiated USC cultures (uUSC) and USC cultures after 5 days of chondrogenic induction from three individual donors. Aggregated cellular structures in induced cultures showed particularly prominent collective E-cadherin membrane staining. Nuclei were counterstained with DAPI. Scale bar, 100 um

To resolve the transcriptional dynamics underlying USC differentiation, we performed integrated single-cell RNA sequencing of undifferentiated and chondrogenically induced USCs. Joint embedding revealed a continuous transcriptional landscape spanning both conditions, with cells distributed along a continuum rather than forming discrete, condition-specific clusters (Fig. 3A, B). Quality control and filtering of the chondrogenically induced dataset are shown in Supplementary Fig. S1C, D.

To infer candidate differentiation trajectories, we applied pseudotime analysis to the integrated dataset [53]. Trajectory orientation was rooted in a uUSC-dominant proliferative cluster, consistent with an early differentiation state. Cells were ordered along an inferred trajectory from undifferentiated populations towards differentiated states (Fig. 3C). Because the cultures contained non-epithelial populations, we tested whether this inferred trajectory reflected differentiation-related structure rather than a mixture of transcriptional states by excluding off-trajectory, non-epithelial clusters and completely re-integrating, re-clustering and re-embedding the remaining epithelial-derived subset before re-running pseudotime (Additional file 1, Supplementary Fig. S4; Additional file 4). The coarse direction from undifferentiated uUSC towards cartilage-like states was preserved and became biologically more consistent, in that the cartilage atlas-aligned endpoints now occupied the latest pseudotime positions. However, the detailed pseudotemporal ordering was not robust (multiple lineages were recovered and the cluster-level rank correlation with the original ordering was only approximately 0.5 across independent reanalyses). We therefore interpret pseudotime qualitatively and as hypothesis-generating, and refrain from quantitative pseudotime claims. Along this inferred trajectory, proliferation-associated genes such as *TOP2A* declined, whereas a candidate intermediate transcriptional state marked by increased *CDH1* expression emerged (Fig. 3D–F). This *CDH1* expression rose to an early peak along the non-robust pseudotime axis and decreased thereafter, so that *CDH1* was not sustained at later pseudotime (Fig. 3D–F; Additional file 1, Supplementary Fig. S2; Additional file 4). This decrease is compatible with transient behaviour; however, because pseudotime is not a true time axis and its fine-grained ordering was not robust, and because *CDH1* expression was sharply cluster-restricted (the large majority of *CDH1*-positive cells confined to two early clusters), the same data are equally compatible with a distinct *CDH1*-positive subpopulation within the heterogeneous culture. We therefore describe this *CDH1*-positive, epithelial-like state as a candidate transient intermediate and explicitly retain the distinct-subpopulation alternative as an equally plausible interpretation that our cross-sectional, single-donor design cannot resolve. To systematically assess markers associated with intermediate transcriptional states, we examined genes enriched in mid-pseudotime clusters. A broader curated marker overview across pseudotime-ordered clusters is provided in Supplementary Fig. S3A. Among these, *CDH1* emerged as a prominent feature of this candidate transitional phase, supporting its role as a marker of an intermediate transcriptional state rather than a constitutive lineage marker. Given the canonical role of *CDH1* in mediating epithelial cell-cell adhesion, its transient upregulation is consistent with the emergence of an epithelial-like aggregation programme during USC differentiation [68, 69]. This interpretation is consistent with the formation of compact cellular aggregates observed during differentiation. Along the re-integrated epithelial pseudotime axis, CDH1 rose to an early peak and decreased thereafter (peak-to-late ratio ≈ 4.5 × ; Spearman ρ ≈ − 0.22), whereas the proliferation marker TOP2A declined monotonically (ρ ≈ − 0.53), consistent with a candidate transient rather than terminal CDH1 state (Fig. 3E, F).

To assess this intermediate state at the protein level, we performed immunofluorescence staining for E-cadherin in independently cultured USC samples from three donors after 5 days of chondrogenic induction. At this stage, aggregated cellular structures displayed particularly prominent E-cadherin membrane staining, consistent with increased epithelial-like cell–cell adhesion during an aggregation-associated intermediate phase (Fig. 3G).

Together, these findings support the hypothesis that, in this donor, USC differentiation is not a direct transition from proliferative to differentiated states but instead may involve a candidate CDH1-associated intermediate transcriptional state. This candidate state likely reflects an epithelial-like aggregation programme associated with subsequent inferred trajectory progression and tissue formation.

### Only a minority of chondrogenically induced USCs map confidently to mature cartilage states in a human reference atlas

**Fig. 4:**
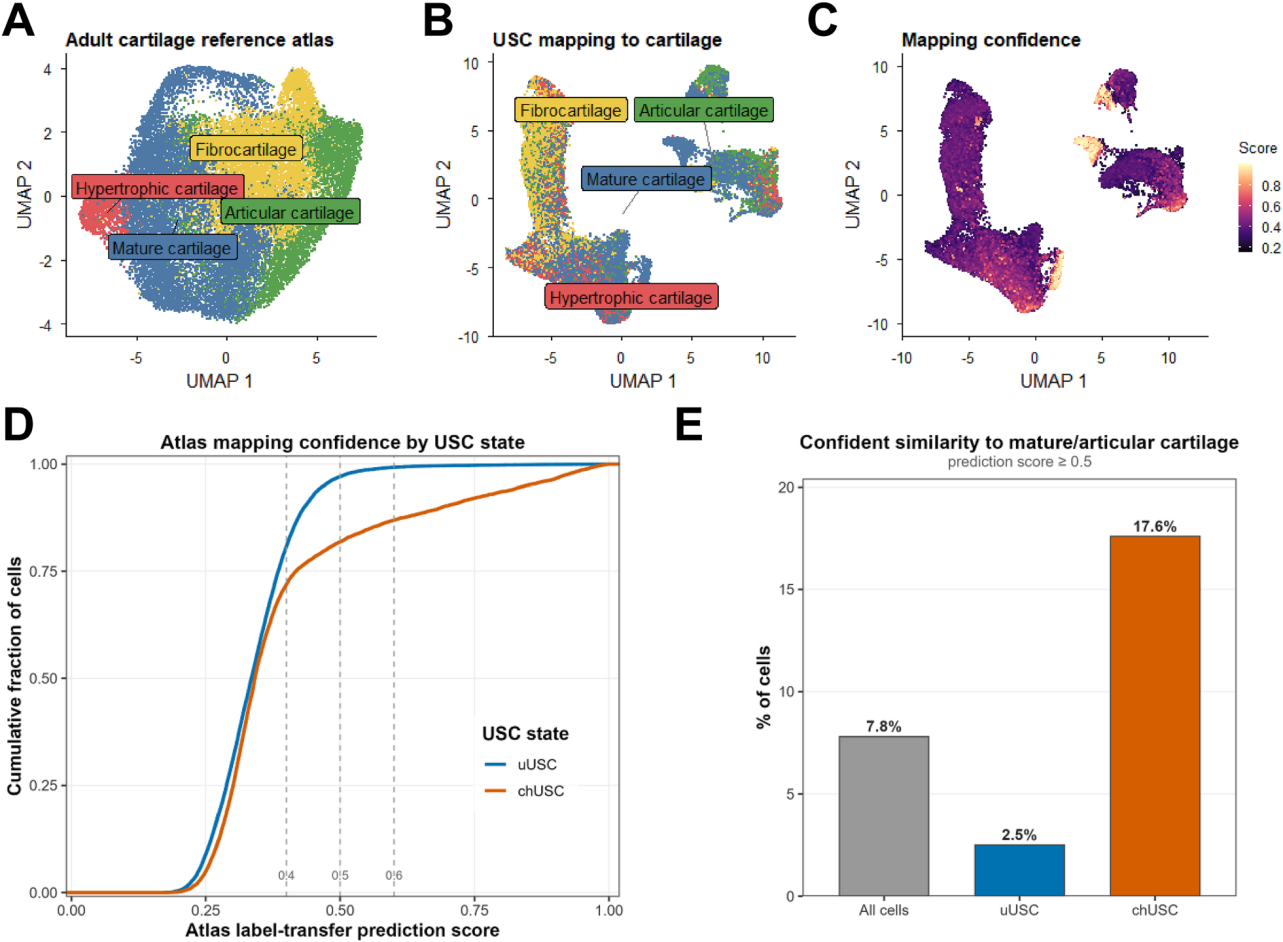
Probabilistic mapping of USCs to a human cartilage reference atlas: only a minority of cells confidently resemble mature cartilage states. **A** UMAP of the human cartilage reference atlas annotated by cartilage state. **B** UMAP of the integrated USC dataset colored by transferred cartilage reference state. Labels denote the best-matching reference state from label transfer, not a validated cell identity. **C** UMAP of the integrated USC dataset showing per-cell mapping confidence (PredictionScore). **D** Empirical cumulative distribution of per-cell prediction scores by stage (uUSC vs chUSC), with thresholds at 0.4, 0.5 and 0.6 indicated; the mass of the distribution lies below 0.4, indicating predominantly low mapping confidence. **E** Fraction of cells with confident similarity (prediction score ≥ 0.5) to mature/articular cartilage reference states: 7.8% of all cells, 17.6% of chUSCs and 2.5% of uUSCs (Additional file 4)

To determine the transcriptional identity of differentiated USCs in a physiological context, we mapped integrated USC datasets onto a human cartilage reference atlas comprising distinct chondrocyte states [48] (Fig. 4A, B). Reference clusters were annotated into articular, mature, hypertrophic, and fibrocartilage states based on established gene expression programmes.

Across all integrated cells, label-transfer confidence was low (median prediction score 0.34; Fig. 4C), and assigning each cell to its single best-matching reference state (argmax, without a confidence threshold) labelled only approximately 40% of cells as cartilage-associated. Because such best-label assignments do not establish identity, we instead quantified the fraction of cells with confident similarity (prediction score ≥ 0.5) to mature/articular cartilage states. By this criterion, only a minority of cells resembled mature/articular cartilage: 7.8% of all cells, 17.6% of chondrogenically induced USCs (chUSCs) and 2.5% of undifferentiated USCs (uUSCs) (Fig. 4E; Additional file 4). Named cartilage-state percentages are not reported, because best-label assignment of each cell to a single named cartilage state would overstate categorical identity at the observed low mapping confidence; mapping is instead summarised probabilistically using per-cell prediction scores.

This confident similarity was concentrated in a few clusters at the differentiated end of the trajectory (notably the cartilage atlas-aligned endpoint clusters), rather than being distributed across the differentiated population (Additional file 1, Supplementary Fig. S3D; Additional file 4). Importantly, label-transfer confidence did not equate with chondrocyte identity: one of the most confidently mapping clusters corresponded to an off-trajectory, neuronal-like population (expressing markers such as *SNAP25* and *CNTN6*) that was excluded from the trajectory analysis, illustrating that high prediction scores can arise even for clearly non-chondrocyte states.

To assess the robustness of these assignments, we examined the full distribution of per-cell prediction scores across stage and clusters (Fig. 4D; Additional file 1, Supplementary Fig. S3B,C). The mass of the distribution lay below 0.4, and the minority interpretation was stable across prediction-score thresholds of 0.4, 0.5 and 0.6. Even among chUSCs, the large majority of cells (more than 80%) did not resemble any reference state confidently, underscoring that confident similarity to mature cartilage was the exception rather than the rule.

Together, these findings indicate that chondrogenic induction increased the proportion of cells with transcriptional similarity to mature/articular cartilage, but that this resemblance remained partial and was confined to a minority of cells at the differentiated end of the trajectory, rather than reflecting a wholesale shift of the culture towards a mature cartilage identity. For cartilage-regenerative applications, this implies that the current chondrogenic USC output is transcriptionally, for the most part, pre-/para-chondrocytic, with purity and maturity as limiting factors; strategies such as enrichment of the atlas-aligned subpopulations, longer or optimised differentiation and multi-donor validation would be required before translational use. This residual heterogeneity was explored further in subsequent analyses.

### Differentiated USCs exhibit heterogeneous lineage-associated programmes including hypertrophic, fibrocartilage-like, and contractile states

**Fig. 5:**
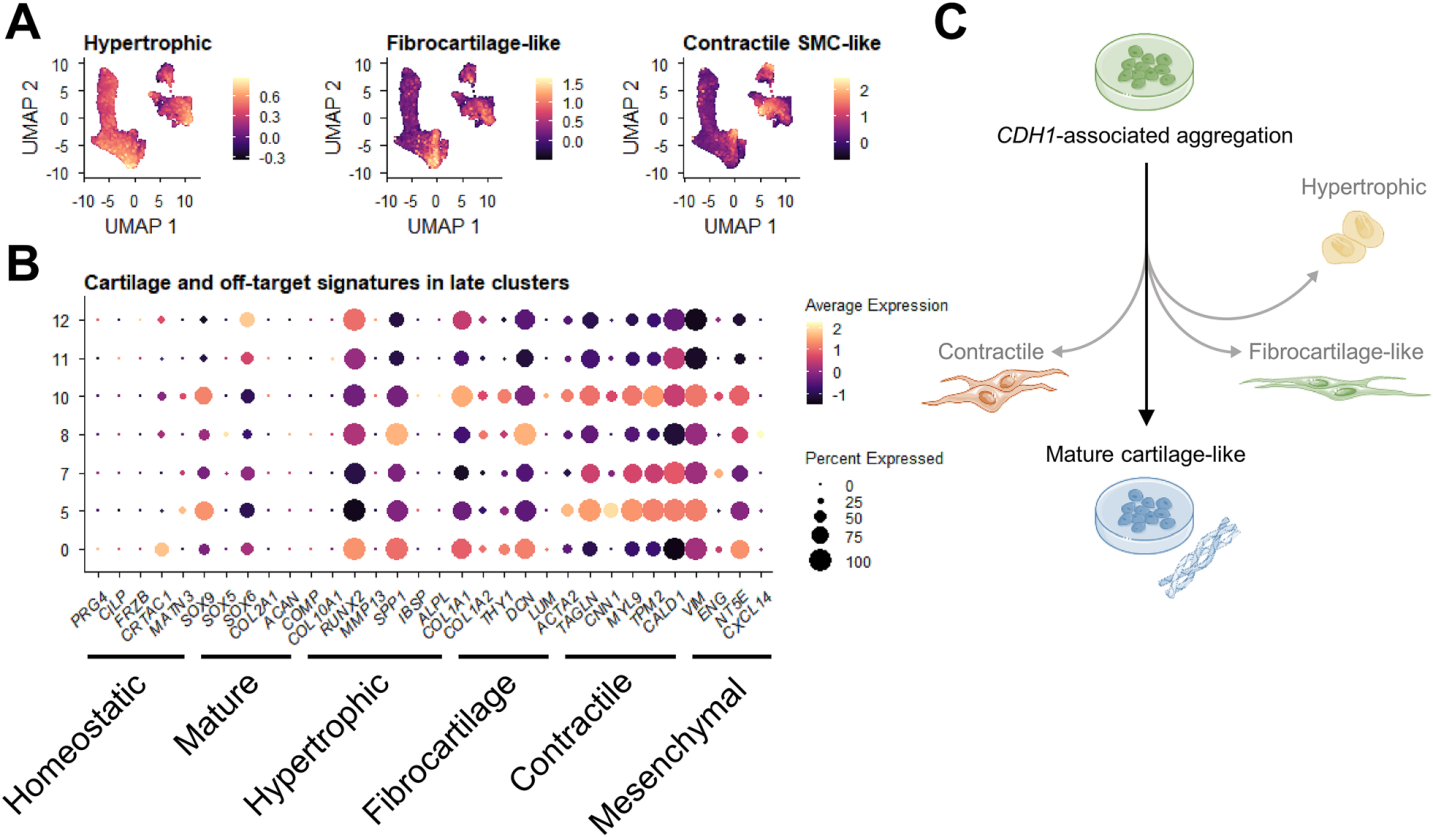
Differentiated USCs exhibit heterogeneous lineage-associated transcriptional programmes. **A** Feature plots showing module scores for hypertrophic, fibrocartilage-like, and smooth muscle/contractile programmes in the integrated USC dataset. **B** Dot plot of cartilage-associated and alternative lineage-associated markers across differentiated/relevant integrated clusters. **C** Schematic summary of the proposed, hypothesis-generating differentiation model, in which USCs may pass through a candidate shared intermediate state before diverging into cartilage-like, hypertrophic-like, fibrocartilage-like, and contractile transcriptional endpoints; only a minority of cells reach states confidently resembling mature cartilage

To further characterize the transcriptional heterogeneity of differentiated USCs, we examined lineage-associated gene programmes across integrated clusters with a focus on late pseudotime states (Fig. 5A, B).

Module scoring revealed the presence of distinct non-cartilage-associated transcriptional programmes within subsets of differentiated cells. In particular, hypertrophic, fibrocartilage-like, and contractile smooth muscle–associated gene signatures were enriched in specific clusters rather than uniformly distributed across the population (Fig. 5A). These programmes were predominantly observed in clusters enriched for chondrogenically differentiated cells, consistent with their emergence during later stages of differentiation.

Cluster-level analysis demonstrated that these lineage-associated programmes were not co-expressed uniformly but instead localized to discrete cellular subpopulations. For example, subsets of clusters exhibited elevated hypertrophic signatures, while others showed enrichment for fibrocartilage-like extracellular matrix genes or contractile smooth muscle–associated markers (Fig. 5B). This partially segregated pattern suggests that differentiated USCs are associated with multiple transcriptional trajectories rather than converging on a single homogeneous endpoint.

Importantly, these alternative programmes were observed alongside, rather than instead of, cartilage-associated gene expression, indicating that USC differentiation gives rise to a spectrum of related but distinct cellular states. This is consistent with the mapping results (Fig. 4), where a fraction of cells aligned with hypertrophic or fibrocartilage reference states despite overall enrichment for mature cartilage.

At the population level, these findings indicate that chondrogenic induction does not result in a uniform mature cartilage phenotype but instead generates a heterogeneous mixture of cartilage-like and related mesenchymal states. This heterogeneity likely reflects the inherent plasticity of USCs and the presence of multiple differentiation trajectories under the applied conditions [67, 70]. These observations may reflect both intrinsic differentiation plasticity and culture-associated effects, which have been reported to influence mesenchymal lineage outcomes in vitro [66, 67].

Together, these results support a model in which USC differentiation proceeds through a shared intermediate phase (Fig. 3) followed by divergence into multiple lineage-associated endpoints, including mature cartilage-like, hypertrophic-like, fibrocartilage-like, and contractile states (Fig. 5C).

### Human serum conditions modulate USC differentiation-associated gene expression and aggregation behaviour

To explore the influence of culture conditions on USC differentiation, we performed exploratory analyses under human serum (HS) conditions in comparison to conventional fetal bovine serum (FBS), including histological and gene expression readouts (Fig. S5).

Under HS conditions, we observed changes in differentiation-associated readouts compared to FBS-based cultures. Alcian blue staining and subsequent quantification indicated reduced glycosaminoglycan deposition under HS conditions (Fig. S5A, B). In contrast, RT-qPCR analysis revealed increased expression of *SOX9* and *CDH1* under HS conditions in both undifferentiated and differentiated samples (Fig. S5C, D).

In addition, HS-cultured cells displayed enhanced cellular aggregation during differentiation, consistent with the increased *CDH1* expression observed at the transcriptional level.

Together, these observations suggest that serum composition may influence USC differentiation-associated transcriptional programmes and cellular behaviour, potentially shifting the balance between aggregation-related processes and extracellular matrix production. However, as these analyses were performed in a limited number of samples, these findings should be considered exploratory and require further validation.

## Discussion

In this study, we combined functional assays with single-cell transcriptomics to resolve the differentiation behaviour of human urine-derived stem cells (USCs) at cellular resolution. Our results indicate that USC differentiation is a continuous and heterogeneous process, characterized by a transient intermediate state and divergent lineage-associated outcomes. Throughout the study, interpretations are based on gene expression programmes and combined marker evidence rather than single-gene readouts.

Functional assays suggested that USCs reproducibly acquire cartilage-associated features under chondrogenic conditions, including glycosaminoglycan-rich extracellular matrix deposition, induction of *SOX9*, and aggrecan-positive matrix formation in 3D culture [59–64]. However, such phenotypic readouts alone do not resolve the underlying cellular heterogeneity, which has been a recurring limitation in the interpretation of mesenchymal differentiation systems [67].

By integrating single-cell RNA sequencing across undifferentiated and differentiated populations from one donor, we inferred a candidate differentiation trajectory rather than a definitive developmental path. Early pseudotime positions were dominated by proliferative cells, while intermediate transcriptional states were characterized by a transient increase in *CDH1* expression as part of a broader aggregation-associated programme. Given the established role of E-cadherin (*CDH1*) in mediating cell-cell adhesion and epithelial organization, this observation is consistent with the emergence of an epithelial-like aggregation-associated state during USC differentiation [71]. While *CDH1* is a prominent marker of this intermediate phase, it should not be interpreted as a lineage-defining factor but rather as one component of a broader transcriptional programme associated with cell-cell adhesion. Similar aggregation-dependent processes have been described in mesenchymal condensation during chondrogenesis, where cell-cell interactions contribute to lineage progression and tissue formation [72–74]. Cadherins are dynamically and transiently regulated during chondrogenic condensation, providing a precedent for transient cadherin dynamics within cartilage-forming programmes; in that developmental setting, however, the transiently and biphasically regulated cadherin is N-cadherin, whereas E-cadherin (*CDH1*) is more sustained, so this precedent contextualises rather than proves our candidate *CDH1* intermediate.

Importantly, probabilistic mapping to a human cartilage reference atlas indicated that only a minority of chondrogenically induced USCs acquired confident transcriptional similarity to mature/articular cartilage states, with overall mapping confidence being low and confident similarity confined to a few clusters at the differentiated end of the trajectory. Best-label assignments alone, which nominally labelled a larger fraction of cells as cartilage-associated, should not be read as identity, as illustrated by an off-trajectory, neuronal-like cluster that mapped with high confidence despite clearly non-chondrocyte markers. The mapped USC states therefore reflect partial transcriptional alignment with, rather than full equivalence to, mature chondrocytes, and a fraction of cells aligned with alternative cartilage-related states, including hypertrophic and fibrocartilage-like populations. In addition, these alternative transcriptional programmes may reflect both intrinsic differentiation plasticity and culture-associated effects, which have been reported to influence lineage outcomes in vitro [67].

Consistent with this observation, module-based analysis revealed the presence of multiple lineage-associated programmes within differentiated populations. Hypertrophic, fibrocartilage-like, and contractile gene signatures were enriched in distinct subsets of cells, indicating that USC differentiation does not converge on a single homogeneous endpoint but instead gives rise to a spectrum of related states.

Together, these findings are consistent with a hypothesis-generating model in which, in this donor, USC differentiation may pass through a candidate shared intermediate transcriptional state, followed by association with multiple lineage-associated trajectories. Within this framework, the *CDH1*-associated state is a candidate transient aggregation phase that may be associated with subsequent lineage progression without defining a terminal identity; equally, given its sharply cluster-restricted expression, it may represent a distinct *CDH1*-positive subpopulation within the heterogeneous culture, a distinction that our cross-sectional, single-donor data cannot resolve.

Exploratory analyses under human serum conditions further indicated that culture conditions may influence differentiation-associated gene expression and cellular behaviour. Increased expression of *SOX9* and *CDH1*, together with enhanced aggregation, suggest that serum composition may modulate differentiation dynamics, although these observations require validation in larger cohorts.

This study has several limitations that should be considered when interpreting the findings. The single-cell analysis was performed on cells derived from a single donor, and inter-individual variability in USC composition and differentiation potential has been reported [13, 28]. In addition, functional validation was restricted to selected markers and phenotypic assays, and further lineage tracing or perturbation experiments would be required to establish causal relationships between intermediate states and differentiation outcomes. These limitations reflect both the experimental scope of the current study and broader challenges in modelling human chondrogenesis in vitro.

Furthermore, mapping to reference atlases provides a probabilistic framework for cell identity assignment but does not demonstrate full functional equivalence to native cartilage [75]. Finally, analyses under human serum conditions were limited to a small number of samples and should be interpreted as exploratory. From a translational perspective, our single-donor findings are hypothesis-generating: if confirmed across independent donors, they would support the potential of USCs as an accessible and non-invasive cell source for cartilage regeneration, but they also show that only a minority of the differentiated culture confidently resembles mature cartilage, so that differentiation purity and maturity, together with control of heterogeneity, remain key hurdles. The observed sensitivity to culture conditions further suggests opportunities to guide USC differentiation through defined or xeno-free systems.

## Conclusions

In conclusion, this single-donor proof-of-concept study provides a hypothesis-generating, single-cell resolution framework for USC differentiation. It nominates a candidate transient, aggregation-associated transcriptional state and documents substantial transcriptional heterogeneity, while showing that only a minority of differentiated cells confidently resemble mature cartilage. Because these observations are based on one donor and on trajectory and mapping inferences whose fine-grained structure is not fully robust, they should be regarded as hypotheses to be tested in independent, multi-donor cohorts rather than as established conclusions. More broadly, these results underscore the importance of resolving intermediate and heterogeneous states, and of interpreting differentiation systems at the level of transcriptional programmes and cellular states rather than individual marker genes.

## Supplementary Information

Additional file 1.

Additional file 2.

Additional file 3.

Additional file 4.

## Data Availability

All sequencing data generated in this study have been deposited in the Gene Expression Omnibus (GEO) database. scRNA-Seq of USCs: accession code GSE307232. Bulk-RNA-Seq of USCs: accession code GSE306729. All the R codes used to generate the results of this study are publicly available on GitHub: https://github.com/alexschulzcell/USC-paper. A preprint of this work has been deposited on bioRxiv (https://doi.org/10.1101/2025.09.26.678723). This preprint has not undergone peer review.

## Abbreviations

ACAN: Aggrecan
BSA: Bovine serum albumin
chUSC: Chondrogenically differentiated urine-derived stem cell
*CDH1*: Cadherin-1
CytoTRACE2: Cellular Trajectory Reconstruction Analysis using gene Counts and Expression 2
DEG: Differentially expressed gene
DMEM: Dulbecco’s modified Eagle medium
DTL: Distal thin limb-like
ECM: Extracellular matrix
FBS: Fetal bovine serum
FGF2: Fibroblast growth factor 2
GAG: Glycosaminoglycan
HS: Human serum
IF: Immunofluorescence
IGF1: Insulin-like growth factor 1
KSFM: Keratinocyte serum-free medium
PBS: Phosphate-buffered saline
PCA: Principal component analysis
PEC: Parietal epithelial cell
PT: Proximal tubule-like
qPCR: Quantitative polymerase chain reaction
RNA-seq: RNA sequencing
RQ: Relative quantification
scRNA-seq: Single-cell RNA sequencing
SCT: SCTransform
TAL: Thick ascending limb-like
TGF-β1: Transforming growth factor beta 1
uUSC: Undifferentiated urine-derived stem cell
UMAP: Uniform manifold approximation and projection
USC: Urine-derived stem cell
