# Supplementary material for: A single-donor proof-of-concept single-cell analysis maps heterogeneous differentiation trajectories toward cartilage-like states in human urine-derived stem cells
Source: Stem Cell Res Ther. 2026 Aug 12;17:276. doi: 10.1186/s13287-026-05223-x (PMC13463475; doi:10.1186/s13287-026-05223-x)
Supplement: Supplementary file 1 [file 13287_2026_5223_MOESM1_ESM.docx]

**Additional file 1: Supplementary Figures**

**A single-donor proof-of-concept single-cell analysis maps heterogeneous differentiation trajectories toward cartilage-like states in human urine-derived stem cells**

Alexander Schulz^1^, Emily M. Brockmann^1^, Miriam Zentgraf^2^, Andreas S. Baur^2^, Steffen Uebe^1^, Arif B. Ekici^1^, Mark Dedden^3^, Sebastian Zundler^3,4^, Christian T. Thiel^1*^

^1^Institute of Human Genetics, University Hospital Erlangen, FAU Erlangen-Nürnberg, Erlangen, Germany

^2^Department of Dermatology, University Hospital Erlangen, FAU Erlangen-Nürnberg, Erlangen, Germany

^3^Department of Medicine 1, University Hospital Erlangen and Friedrich-Alexander-University Erlangen-Nürnberg, Erlangen, Germany

^4^Deutsches Zentrum Immuntherapie (DZI), University Hospital Erlangen, Erlangen, Germany

**Supplementary Figure S1. Quality control and filtering of uUSC and chUSC single-cell datasets.**

(A) Violin plots showing quality control metrics for the undifferentiated USC dataset, including detected genes, UMI counts, and mitochondrial read fraction.
(B) Density plots showing filtering thresholds applied to the undifferentiated USC dataset.
(C) Violin plots showing quality control metrics for the chondrogenically differentiated USC dataset.
(D) Density plots showing filtering thresholds applied to the chondrogenically differentiated USC dataset.

**Supplementary Figure S2. CDH1 dynamics along pseudotime, clusters and stage on the re-integrated epithelial subset.**

(A) Mean *CDH1* and *TOP2A* expression across pseudotime deciles; *CDH1* rises to an early peak (second decile) and decreases thereafter (peak-to-late ratio ≈ 4.5×; non-monotonic, Spearman ≈ −0.22), whereas *TOP2A* declines monotonically (Spearman ≈ −0.53) as an early proliferation marker.
(B) Percentage of *CDH1*-positive cells across pseudotime deciles.
(C) *CDH1* expression across the re-integrated clusters (ordered by pseudotime); *CDH1* is sharply restricted to two early clusters that contain the large majority of *CDH1*-positive cells, a discrete distribution compatible with either a transient intermediate or a distinct *CDH1*-positive subpopulation.
(D) *CDH1* expression by stage (uUSC vs chUSC). Pseudotime is not a true time axis and its fine ordering is not robust (Supplementary Fig. S4); these data are interpreted as hypothesis-generating in a single donor.

**Supplementary Figure S3. Supplementary marker and mapping-confidence analyses.**

(A) Curated marker overview across pseudotime-ordered integrated USC clusters, including early proliferative, *CDH1*-associated intermediate, and alternative lineage-associated programmes.
(B) Violin plot of mapping confidence (PredictionScore) in undifferentiated and chondrogenically differentiated USC populations.
(C) Violin plot of mapping confidence (PredictionScore) across integrated USC clusters.
(D) Fraction of cells per integrated cluster that map confidently (prediction score ≥ 0.5) to mature/articular cartilage reference states. Confident mapping is concentrated in a few clusters at the differentiated end of the trajectory; notably, an off-trajectory, neuronal-like cluster (cluster 12, excluded from the trajectory analysis) also maps with high confidence, illustrating that label-transfer confidence does not equal chondrocyte identity.

**Supplementary Figure S4. Robustness of the pseudotime reanalysis on the re-integrated epithelial subset.**

(A) Pseudotime distribution by stage (uUSC vs chUSC) on the re-integrated epithelial subset, computed after exclusion of non-epithelial, off-trajectory clusters (integrated clusters 5, 10 and 12); the coarse direction from undifferentiated to differentiated states is preserved.
(B) Pseudotime distribution across the re-integrated clusters, ordered by median pseudotime; the cartilage atlas-aligned endpoint clusters now occupy the latest positions.
(C) Per-cell comparison of re-integrated versus original pseudotime (Spearman ≈ 0.58; cluster-median rank Spearman ≈ 0.53) and
(D) re-integrated versus an independent subset analysis on the existing embedding (Spearman ≈ 0.57). The recovery of multiple lineages and only moderate rank correlations indicate that the coarse trajectory direction is robust whereas the fine-grained pseudotemporal ordering is not; the trajectory is therefore interpreted qualitatively and as hypothesis-generating.

**Supplementary Figure S5. Exploratory comparison of FBS- and HS-based differentiation conditions.**

(A) Alcian blue staining of FBS- and HS-based chondrogenic monolayer cultures. Representative images illustrate reduced glycosaminoglycan staining and increased aggregation under HS conditions.
(B) Quantification of Alcian blue staining normalized to DNA content, indicating reduced glycosaminoglycan deposition under HS conditions compared with FBS (OD650/DNA in FBS = 0.010 ± 0.0003; OD650/DNA in HS = 0.006 ± 0.0004; *n* = 4; unpaired *t*-test, *p* < 0.0001).
(C) RT-qPCR analysis of *SOX9* expression under FBS and HS conditions in both undifferentiated USC cultures and chondrogenic pellet cultures (chUSC). In undifferentiated samples, HS was associated with higher *SOX9* expression than FBS (mean normalized RQ: FBS = 0.472, HS = 1.113; *n* = 3 per group; Welch’s *t*-test, *p* = 0.00035). In chondrogenic samples, HS was likewise associated with higher *SOX9* expression than FBS (mean normalized RQ: chFBS = 0.611, chHS = 1.389; *n* = 3 per group; Welch’s *t*-test, *p* = 5.45 × 10^-5).
(D) RT-qPCR analysis of *CDH1* expression under FBS and HS conditions in both undifferentiated USC cultures and chondrogenic pellet cultures. In undifferentiated samples, HS was associated with higher *CDH1* expression than FBS (mean normalized RQ: FBS = 0.0137, HS = 0.0598; *n* = 3 per group; Welch’s *t*-test, *p* = 0.0014). In chondrogenic samples, HS was likewise associated with higher *CDH1* expression than FBS (mean normalized RQ: chFBS = 0.184, chHS = 1.816; *n* = 3 per group; Welch’s *t*-test, *p* = 1.19 × 10^-4). Bars with undetermined Ct values or values exceeding 40 cycles were indicated as ND where applicable.
